# Staying Strong Toolbox: Co-design of a physical activity and lifestyle program for Aboriginal families with Machado-Joseph disease in the Top End of Australia

**DOI:** 10.1371/journal.pone.0244311

**Published:** 2021-02-05

**Authors:** Jennifer J. Carr, Joyce Lalara, Gayangwa Lalara, Gwen Lalara, Bronwyn Daniels, Alan R. Clough, Anne Lowell, Ruth N. Barker

**Affiliations:** 1 College of Healthcare Sciences, James Cook University, Cairns, Queensland, Australia; 2 MJD Foundation, Darwin, Northern Territory, Australia; 3 Community-based Health Promotion and Prevention Studies Group, Medical and Veterinary Sciences and Australian Institute of Tropical Health and Medicine, College of Public Health, James Cook University, Cairns, Queensland, Australia; 4 Northern Institute, Charles Darwin University, Darwin, Northern Territory, Australia; Institute of Mental Health, SINGAPORE

## Abstract

Physical activity has positive health implications for individuals living with neurodegenerative diseases. The success of physical activity programs, particularly in culturally and linguistically diverse populations, is typically dependent on their alignment with the culture, lifestyle and environmental context of those involved. Aboriginal families living in remote communities in the Top End of Australia invited researchers to collaborate with them to co-design a physical activity and lifestyle program to keep individuals with Machado-Joseph disease (MJD) walking and moving around. The knowledge of Aboriginal families living with MJD, combined with findings from worldwide MJD research, formed the foundation for the co-design. An experience-based co-design (EBCD) approach, drawing from Indigenous and Participatory methodologies, was used. An expert panel of individuals with lived experience of MJD participated in a series of co-design phases. Prearranged and spontaneous co-design meetings were led by local community researchers within each phase. Data was collected using a culturally responsive ethnographic approach and analysed thematically. Sixteen panel members worked to develop the ‘Staying Strong Toolbox’ to cater for individuals with MJD who are ‘walking strong’; or ‘wobbly’; or ‘in a wheelchair’. Based on the ‘Staying Strong Framework’, the Toolbox was developed as a spiral bound A3 book designed to guide the user to select from a range of activities to keep them walking and moving around and to identify those activities most important to them to work on. The ‘Staying Strong Toolbox’ is a community driven, evidence based resource for a physical activity and lifestyle program for Aboriginal families with MJD. The Toolbox provides a guide for health professionals and support workers to deliver person-centred support to Aboriginal families with MJD, and that can be modified for use by other families with MJD or people with other forms of ataxia around the world.

## Introduction

The benefits of physical activity for individuals with neurodegenerative conditions are well known [1–4]. Physical activity has been shown to enhance function and well-being and appears to have neuroprotective effects for those with neurodegenerative diseases [1, 2, 5–8]. Yet, one size does not fit all, in terms of types of physical activities that are most effective, and the appropriate dosage [9]. Individuals with neurodegenerative diseases live with a variety of impairments, and experience considerable changes in function, often over a 20-year period [10–13]. Tailored physical activity interventions that suit the lives of individuals, their environment, interests, responsibilities and available resources, seem to reap the most rewards for mobility and adherence to physical activity [9, 14, 15]. Flexibility within these interventions to change as their disease progresses is also important [15].

MJD is an autosomal dominant neurodegenerative disease that leads to ataxia and progressive decline in motor function [11]. Most people with MJD are wheelchair bound and dependent on support for activities of daily living within 10 years of symptom onset [16, 17]. MJD is the most common spinocerebellar ataxia (SCA) worldwide [18], and appears to be most prevalent in affected Aboriginal communities in the Top End of Australia [12, 19–22]. Aboriginal families of Groote Eylandt, in Ngukurr and related Aboriginal communities have experienced the devastating impact of MJD for generations [23]. Their culture and lifestyle vary considerably compared to families in other regions of the world [12, 24].

It is important to recognise that acceptability and effectiveness of physical activity programs in culturally and linguistically diverse populations is dependent on whether harmony exists between the program and the culture, lifestyle, geographical and environmental context of those involved [12, 25, 26]. In essence, physical activity is only likely to be beneficial for Aboriginal families with MJD, if it is relevant, meaningful and culturally responsive [12, 27, 28]. Accordingly, methodologies such as experience-based co-design (EBCD) are required for program development to ensure end-users are actively involved in the process from beginning to end [29]. Recognised in Indigenous research contexts, EBCD fosters ongoing input from communities to lead the design, development and evaluation of programs, to ensure they are meaningful and relevant in accordance with their priorities [30–32].

Families with MJD from Groote Eylandt and Ngukurr, supported by the MJD Foundation, recently partnered with university researchers to explore the best ways to keep walking and moving around [12]. The objective of the partnership was to bring together the knowledge of families with MJD [12], with what is known from MJD research from around the world [33], to develop a physical activity program [33]. Based on the findings of two earlier studies [12, 33], the aim of this current study was to co-design a meaningful physical activity and lifestyle program tailored to the priorities of Aboriginal families with MJD in the Top End of Australia.

## Methods

### Study design

An experience-based co-design (EBCD) approach embedded within Indigenous and Participatory methodologies was used to ensure dominance of the views of Aboriginal families with MJD, their communities and community researchers [31, 34, 35]. EBCD was chosen because it is an approach which fosters engagement of individuals, communities and health care professionals as active participants in the development of services for their use in the future [36]. EBCD provided a guiding framework to allow families with MJD as experts, by lived experience, to develop a physical activity and lifestyle program [29, 31, 36]. The EBCD Australia Toolkit [37], recommendations for codesign studies [38] and the six stages of the EBCD cycle [29, 39] were drawn upon to guide our co-design process. The Consolidated Criteria for Reporting Qualitative Research (COREQ) guidelines were used to document the process [40]. Ethical approval was granted prior to study commencement by the Human Research Ethics Committee (HREC) of the Northern Territory (NT) Department of Health and Menzies School of Health Research (HREC 2018–3044) and externally approved by James Cook University HREC (H7367). Permission was granted to conduct the research from Anindilyakwa Land Council and Northern Land Council and appropriate land permits were secured prior to research commencement. Participants in this study have provided written informed consent (as outlined in PLOS consent form) for their images to be used.

### Setting

The Aboriginal people of the Groote Eylandt Archipelago (Warnumamalya) have occupied their lands for around eight thousand years [41]. Approximately ~1500 Warnumamalya occupy the Aboriginal communities of Angurugu, Umbakumba and Amakalyakba (Bickerton Island) within the Groote Eylandt Archipelago [22]. Anindilyakwa is the main language spoken [41]. On the mainland to the west (approximately 218km in a direct line), live the Aboriginal people of Ngukurr and Urapunga who have occupied the Roper Gulf region for 40,000 years. The major language spoken in Ngukurr is Kriol as well as Ngalakan, Alawa, English and other Aboriginal languages [42]. Approximately 972 Aboriginal people live in Ngukurr today [43].

**Fig 1:**
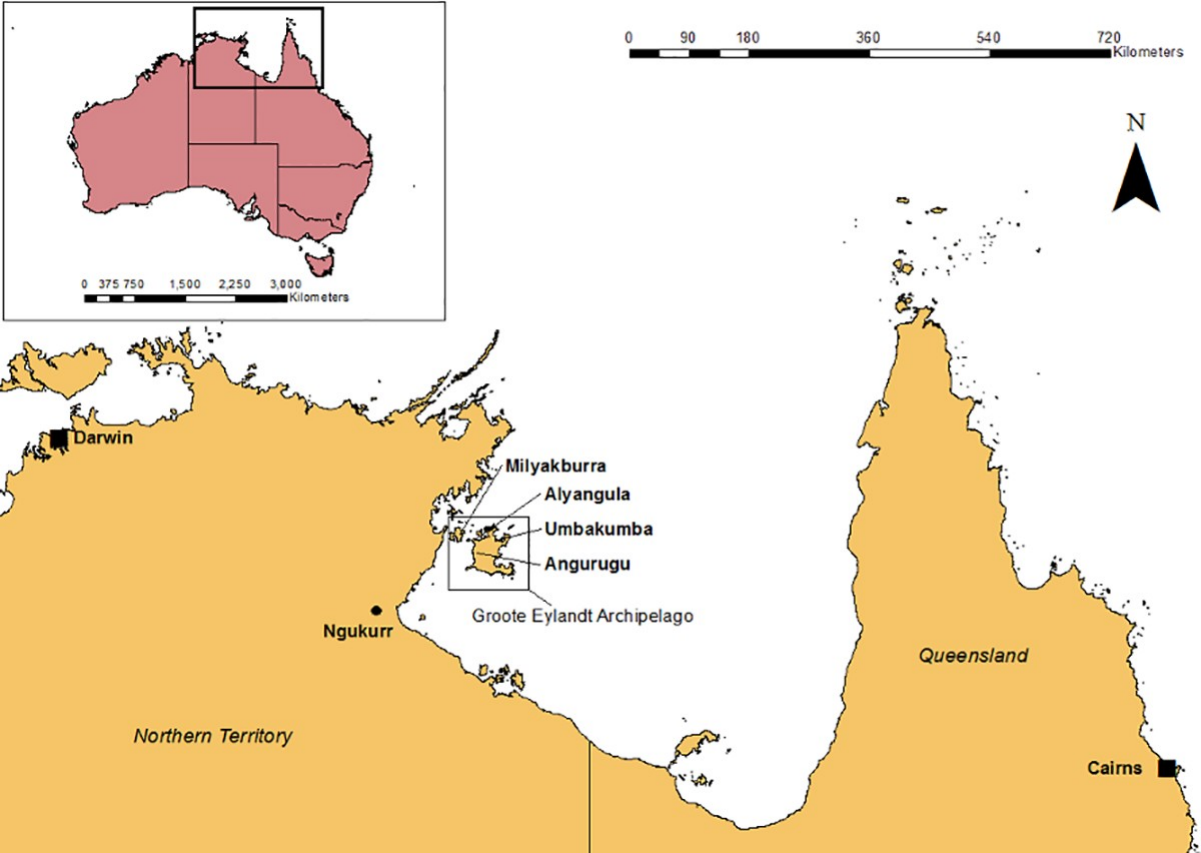
Localities of the Groote Eylandt Archipelago and Ngukurr. Map created with ESRI ArcGIS using Australian Bureau of Statistics (ABS) data [48].

Like many Aboriginal communities in the Top End, families maintain traditional systems of social organisation and cultural practices that include ceremonial responsibilities, fishing, hunting and gathering [41, 44]. Both Ngukurr and the Groote Eylandt Archipelago are considered very remote regions of Australia [41, 45] (Fig 1). Each has a general store, school, health clinic, emergency services post and day-based aged care centers (no 24 hour care available) [42, 46]. The closest tertiary hospital is at least 650km away. Aboriginal families with MJD in both areas are supported by the MJD Foundation. The MJD Foundation is a community driven organisation founded on Groote Eylandt that works in partnership with Aboriginal Australians, their families and communities living with MJD to provide comprehensive supports and engage in research, providing hope for the future [47].

Families in these very remote Top End communities face daily challenges in relation to limited access to carer support, health services, infrastructure and accessible housing. Harsh environmental factors, including extreme heat, monsoonal wet seasons and rough terrain add to the complexity. As their function declines, many individuals with MJD need to move to the city hundreds of kilometres away for medical or residential care, facing cultural and linguistic isolation, away from their families, communities and homelands.

### Research team

The research team for this current study consisted of Aboriginal community research partners (CRPs) from Groote Eylandt (JL, GL, GwL) and Ngukurr (BD), who were experienced community workers and cultural advisors either living with MJD or who have a close family member with MJD. Each CRP (JL, GL, GwL and BD) coordinated the research in their respective communities and led meaning-based translation of the work into English. An invited non-Aboriginal researcher (JC), an experienced physiotherapist in neurological rehabilitation, worked alongside families to conduct this research (supported by RB). JC was well known to families with MJD on Groote Eylandt and in Ngukurr through the two earlier studies on which this current study was based [12, 33]. JC partnered with JL, GL, GwL and BD in study preparation, data collection, analysis and manuscript preparation. Non-Aboriginal associate investigators (RB, AL and AC) supervised the research, each of whom brought more than 20 years of experience working and conducting research in remote Aboriginal communities. Senior community members from Groote Eylandt and Ngukurr provided endorsement on completion of each co-design phase.

### Foundation for co-design

**Fig 2:**
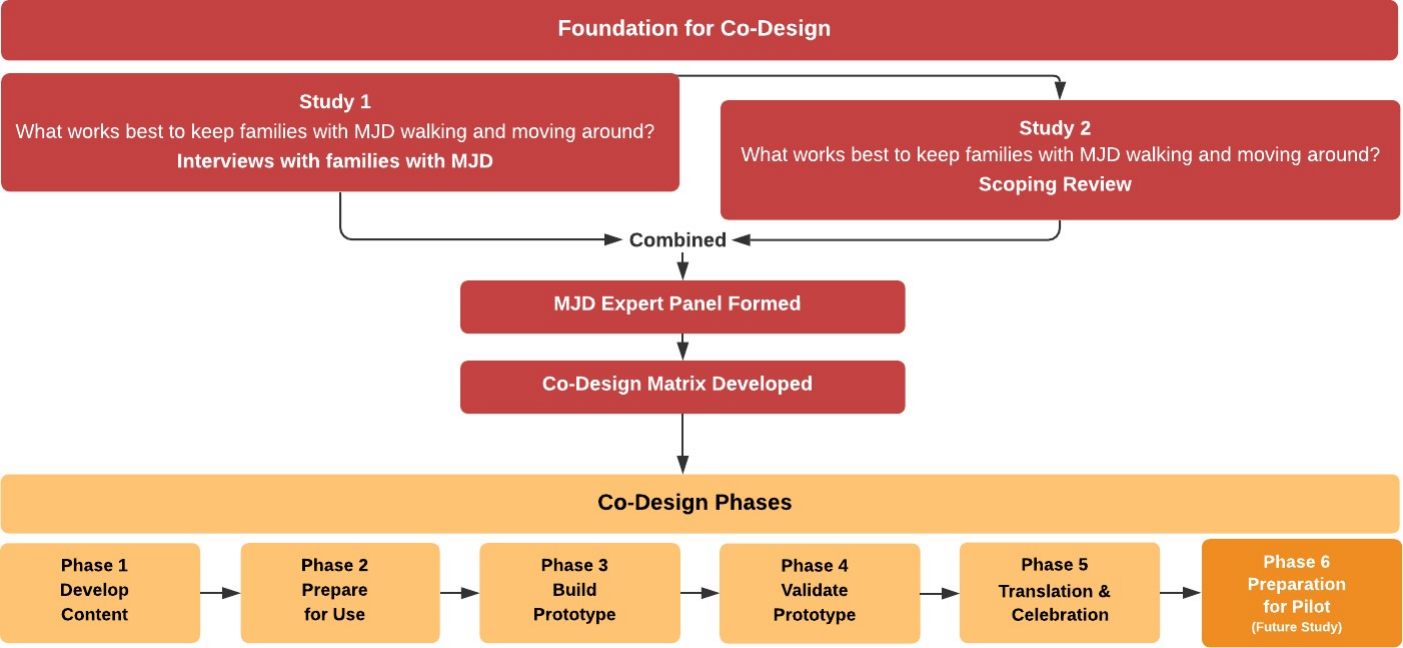
Foundations for co-design process.

The Co-design process is outlined in Fig 2. Two earlier studies formed the foundation for the co-design process [12, 33]. Study 1 involved interviews with individuals and families with MJD to explore ‘what is important’ and ‘what works best’ to keep people with MJD walking and moving around [12]. ‘Walking and moving around’ refers to the ability to move around from place to place, including around the home to do everyday tasks, as well as around the community, and further afar, inclusive of all aspects of mobility. The term was selected by Aboriginal community researchers as it could be translated across multiple Aboriginal languages. Participants emphasised the importance of staying strong on the outside (i.e., physically), and the inside (i.e., mentally, emotionally, spiritually) by ‘exercising your body’, ‘keeping yourself happy’, having ‘something important to do’, ‘going Country’, ‘searching for good medicine’ and ‘families helping each other’. These six domains formed the ‘Staying Strong Framework’ and provided the core principles for the physical activity program [12].

In Study 2, the Staying Strong Framework provided a lens for a scoping review of peer reviewed literature worldwide to map interventions that promote walking and moving for individuals with MJD [33]. Most studies focussed on ‘exercising your body’ (e.g., walking training, balance training or task-specific training) with positive findings when exercise occurred for at least 50 minutes in duration, two to three times each week, for approximately 4 weeks. Some studies assessed medications, but not traditional medicines and no medications could be strongly recommended. No studies explored the impact on mobility of ‘going Country’ (e.g., spending time in culturally significant places, community participation, outdoor mobility, sport/recreation); ‘families helping each other’ (e.g., the impact or relationship of family support on functional mobility or emotional wellbeing); having ‘something important to do’ (e.g., goal orientated, or task specific training based on individual goals/priorities/interests) or ‘keeping yourself happy’ [33].

### MJD Expert Panel

The MJD Expert Panel was formed to co-design the physical activity and lifestyle program. Panel members were purposively selected from Groote Eylandt and Ngukurr. To gain a range of experiences, panel members included males and females of different ages and functional levels, living with MJD and/or carers and/or support workers for those with MJD [12]. Individuals were excluded if they had a moderate to severe intellectual or psychological impairment to ensure informed consent and to reduce participant burden [12]. The research team, exclusive of JC, were included as panel members.

Panel members were recruited by MJD Foundation service providers, by community researchers (JL, GL and BD) or in response to an independent expression of interest to the research team. Potential panel members were informed by the research team about what would be required in each co-design phase. Either written or oral consent was recorded prior to research commencement. Panel members were reimbursed for their participation in each co-design phase with a $75 (AUD) shopping voucher. Community researcher wages were funded through a research grant from the Lowitja Institute for Aboriginal and Torres Strait Islander Research and JC was supported by a federally funded PhD scholarship.

### Co-design matrix

In preparation for Phase 1 co-design meetings, the research team collaborated to combine the findings of the foundational studies into a matrix based on the Staying Strong Framework and three functional levels that families with MJD typically describe (walking strong, walking wobbly, using a wheelchair). The matrix was operationalised in a large book of A3 sized worksheets, to present the findings, guide discussion and collect panel member feedback. Each worksheet page outlined one domain of the Staying Strong Framework (exercising your body, searching for good medicine, families helping each other, keeping yourself happy, something important to do) and included a blank table divided into three functional levels: ‘walking strong’ (i.e., walks independently), ‘walking wobbly’ (i.e., requires physical assistance or a walking aid) or ‘in a wheelchair’ (i.e., wheelchair dependent). The blank table was for panel members to contribute their own ideas about activities to keep ‘walking and moving around’ across each functional level. Prompts were used to guide panel member discussion, such as, “what are the best ways to be ‘exercising your body’ if you are: walking strong? walking wobbly? In a wheelchair’” (Table 1).

**Table 1:** Phase 1 –co-design meeting guide.

Phase 1 Co-Design Meeting Guide	
What are the best ways to be **exercising your body** if you are	Walking strong?
	Walking wobbly?
	In a wheelchair?
What are the best ways to be **going Country** if you are	Walking strong?
	Walking wobbly?
	In a wheelchair?
What are the best ways to be **keeping yourself happy** if you are	Walking strong?
	Walking wobbly?
	In a wheelchair?
What are the best ways to be **searching for good medicine** if you are	Walking strong?
	Walking wobbly?
	In a wheelchair?
What are the best ways for **families to be helping** each other if you are	Walking strong?
	Walking wobbly?
	In a wheelchair?
What are **important things to do** if you are	Walking strong?
How do you find **something important to do**?	Walking wobbly?
	In a wheelchair?

The final page extended on the domain of ‘something important to do’ and was designed to capture the goals of users over a 4-week physical activity period, where participants score themselves using the Patient Specific Functional Scale (PSFS) [49]. The PSFS is a visual analogue scale that ranges from 0 to 10 with 0 representing ‘unable to perform activity,’ and 10 representing ‘able to perform the activity to my satisfaction’, and would be scored before and after the 4-week physical activity period [33].

### Co-design phases

Co-design phases were arranged at time periods throughout the year when all researchers and panel members could participate. Particular attention was paid to avoiding periods of ceremonial and cultural responsibilities. Each phase provided opportunities for multiple co-design meetings to occur, either prearranged or spontaneously, led by JL, GL, GwL and BD in the preferred language of panel members [31]. Prearranged meetings were those meetings scheduled by the research team. Spontaneous meetings were those meetings that occurred when panel members were together for social reasons (e.g. fishing), as part of day-to-day conversations or when they chose to visit the research team for further discussions or feedback. JC spoke English as a first language but endeavoured to learn and converse in the languages spoken in each respective community.

A culturally responsive ethnographic approach was used to gather data [50]. In line with this approach, researchers were immersed within each community to observe panel members within their local context [50]. Panel member discussions were facilitated using a narrative, open ended and informal interviewing style [50]. Details of what occurred in each phase are outlined in Table 2.

**Table 2:** Co-design phases.

Phases	Actions
Phase 1 Develop Content	• Staying Strong Framework and co-design matrix presented to panel.
	• Prompts provided to facilitate suggestions for activities for each functional level.
	• JC scribed while panel deliberated.
	• Panel members reviewed, added or refined activities documented.
	• Research team collated suggested activities into one worksheet.
	• Data coded and categories emerged for each ‘Staying Strong’ domain.
	• Panel members provided a copy of their individual worksheet.
Phase 2 Prepare for Use	• Worksheets reviewed from Phase 1 reviewed and modified by panel members.
	• Prompt questions guided discussion (Table 5).
Phase 3 Build Prototype	• Worksheets collated and developed into a book titled ‘Staying Strong Toolbox’ with tick boxes for each activity (tool).
	• ‘Staying Strong Toolbox’ provided to each panel member.
	• Panel members modified prototype content, look, feel and use.
Phase 4 Validate Prototype	• Trialled use of Toolbox book with panel members with MJD to review ease of use.
	• Home programs compiled for each panel member with MJD based on tools they selected.
	• Issues/changes required discussed with panel members.
Phase 5 Translation and Celebration	• Research discussed with community stakeholders (MJD Foundation staff, health service, media centre, and land council staff) throughout co-design phases from inception to completion.
	• Presented findings at national and international conferences.

Data collected from co-design phases included audio recordings and/or field notes and a research diary kept by JC to record daily interactions within the team and program iterations that panel members felt were required. The research team collaborated to complete meaning-based translation and transcription of meeting data into English as required and all panel members were invited to review, verify or modify transcriptions. As panel members were more able to read and write in English than their respective first languages, their preference was to document information in English. Controlled English (free from culturally specific metaphors, using terms, narratives and sentence structures that were easily translatable) [51] was used to ensure all panel members could participate fully in meetings.

### Data analysis

A qualitative descriptive approach was used to analyse data from panel meetings [52, 53]. Data related to program activities and information were collated according to each domain of the Staying Strong Framework and analysed thematically, through discussions between researchers, using Microsoft Word.

In each co-design phase, panel members were consulted by the research team to identify, discuss and refine key activity categories and themes that emerged. On completion of each co-design phase, findings were discussed with senior community members to verify information gathered and to seek approval to update the prototype.

## Results

A total of 16 MJD Expert Panel members from Groote Eylandt (n = 8) and Ngukurr (n = 8) participated in co-design phases one to five. Characteristics of panel members are included in Table 3.

**Table 3:** Characteristics of MJD Expert Panel Members (n = 16).

Participant Characteristics	Number (%)
Community	
Groote Eylandt	8 (50)
Ngukurr	8 (50)
Gender	
Female	10 (70)
Male	6 (30)
Age	
30–39	3 (15)
40–49	7 (35)
50–59	4 (20)
> 60	2 (10)
MJD	
Yes	10 (50)
No	6 (30)
Mobility Status^a^	/10
Independent	4 (40)
Requires assistance*	3 (30)
Wheelchair dependent	3 (30)
Activities of daily living**	/10
Independent	3 (30)
Requires assistance	7 (70)
Carer of individual with MJD	13 (65)
Community Leader	3 (15)
MJDF Community Worker	5 (25)

Abbreviations

*, Requires physical assistance and/or mobility aid for indoor/outdoor mobility

**, Requires physical assistance or assistive devices; %, percentage

a, MJD Representatives only; MJD, Machado-Joseph disease; MJDF, MJD Foundation.

Co-design phases one to five took place between April 2018 to December 2018 to develop what became known as the ‘Staying Strong Toolbox’. Each phase ranged between two- and five-weeks in duration (mean = 3) during which time the non-Aboriginal researcher remained in each remote community and the relevant community researchers remained in their respective local community to coordinate the research. Meetings occurred at various locations including the beach, homes of panel members, the local aged care centre, in the outdoors on traditional homelands, at the local store or in the back of the research team’s four-wheel drive vehicle. Prearranged meetings included one on one interviews (Groote Eylandt (GTE) = 6; Ngukurr (NGU) = 6), dyadic interviews (GTE = 3; NGU = 5), small groups (GTE = 5; NGU 6) and workshops (GTE = 5; NGU = 5). Spontaneous meetings included interactions via text messages, phone calls, or face to face visits throughout the day or in the evening. Meetings commonly occurred early to mid-morning or in the late afternoon and evening. A total of 41 prearranged meetings occurred (exclusive of spontaneous meetings), 22 in Ngukurr and 19 on Groote. Each meeting was held for between 40 minutes and up to four hours (average ~1.5 hours).

Research grant expenditure of less than $AUD 42,000 covered travel, accommodation, wages, panel member reimbursement, meeting refreshments and technology (tablets for community researchers). JC was not local to each community but spent extended periods of time (3 months) on a number of occasions in each community. Between visits, the research team collaborated via Zoom and telephone.

### Phase 1 –develop content

At the completion of Phase 1, 567 activities and strategies for staying strong were gathered from panel members and categorised under each Staying Strong Domain (Table 4). Statements that were essentially ‘tips for success’ for individuals who may use the program also emerged from panel member discussion.

**Table 4:** Phase 1 –summary of panel member responses.

Staying Strong Domain	Categories	Tips for Success
**Exercising Your Body**	Walking Practice	*“You have to be able to do exercise in a place away from an audience*, *with families or with other MJD people*.*” (4)*
	House Working or Job Working	
	Getting Strong Muscles	
	Doing Sport	*“…It’s better to do exercise with people we trust and that make us happy*.*” (2)*
**Going Country**	Hunting Ideas	*“Sitting too long gives you cramps and makes you wobbly*. *You have to try keep your body moving*.*” (7)*
	Fishing Ideas	
	Walking–Bush or Beach	
**Searching for Good Medicine**	Looking for Medicine Yourself	*“It’s about eating well as well as getting it [bush medicine]… it’s good exercise but it will make you want to go and find more too…*. *Because they are so good to eat…good for you inside and outside*.*” (11)*
	Working with Family to Look for Medicines	
	Bringing Medicine Home to Help Other Families	
	Cooking Medicine Yourself or Drawing on Others	
	Getting Help From the Clinic with Medicines	*“Have lots of good food… the best food comes from the land and saltwater…don’t eat too much greasy food at the shop [local take away store]*.*” (5)*
**Keeping Yourself Happy**	Doing Fun Things	*“…Find things that make you feel good and take your mind off the sickness*, *for anyone with MJD*. *(1)*
	Getting Out of the House	
	Being with Families	
	Staying Happy and Healthy	
	Teaching the Kids About MJD	
**Families Helping Each Other**	Helping Your Family With and Without MJD	*“Help other people with MJD to move their body*, *go Country*, *do things for them*, *support them*. *It will keep you moving your body…” (4)”*
	Helping Yourself	
	How Families Can Help Their Loved Ones With MJD	
**Something Important to Do**	Finding Something Important to Do	*“…Important things keep you busy…take your mind off that sickness*. *Find ways to make things easy so you can do them yourself*. *Find those things you can do by yourself at home*.*” (1)*
	Finding a Job to Do	
	Working Around the House	
	Important Ways to Support Your Family	
	Finding Help to Do Things That Are Important	

Panel members stated photos should be included to provide pictorial representation of the activities suggested. Panel members asked research team members to take their photo whilst undertaking their suggested activities or brought their own photos of themselves doing the activity.

‘We have to show people what we are doing so they might do it too’ (13)

### Phase 2 –prepare for use

Panel members reviewed the worksheets from Phase 1 and provided feedback on repetition of activities, missing information, irrelevant information, and added new activities. In this process, panel members decided that the worksheets should be developed into a book, and called a Toolbox. The book would list the range of activities (i.e., tools in the Toolbox) participants had suggested, aligned with each Staying Strong domain section. Panel members felt that all individuals with MJD, regardless of functional level, could use this book. They could look through the list of tools in each domain of the book, with their families, and tick what tools they thought would suit them best. Their chosen tools (i.e., activities) would form their own Staying Strong Toolbox program to keep themselves strong on the inside and outside. Through further discussions and agreement between participants, ‘Staying Strong Toolbox’, or ‘Toolbox’ in short was introduced as the name for the book. Panel member responses to the prompt questions were collated and are summarised in Table 5.

**Table 5:** Phase 2—summary of panel member responses.

Additional Prompts	Summary of Responses
**What should it look like?**	• Tools (activities) should be listed as tick boxes.
	• Individuals with MJD could tick what tools suit them best.
	• Changes made to pictures, colours, font size, layout, language.
	*“This book has ideas for everyone*. *We’ve all put our ideas into this book*. *These ideas are tools*. *Like when you are trying to fix a car*, *all cars are different and have different problems*, *you need to find the right tool to fix the problem… People have to pick the right tools for themselves they think will help…they can just tick those things*.*”(7)*
**What should we do with the Toolbox?**	• Help families keep walking and moving and slow MJD.
	• Share with families with MJD in other parts of the world.
	• Use to help families to communicate what is important to them.
	○ To their families.
	○ To their health professionals/support workers.
	• Help individuals identify:
	○ What they can do by themselves.
	○ What they require support with.
	• Guide support workers/ health professionals unfamiliar with MJD.
	• Modify it to suit other families in other locations.
	• Share with other families so they may build their own Toolbox.
	*“This Toolbox is for everyone to use*, *no matter if people are strong or using a wheelchair*. *It will help give people ideas on what they can do to keep themselves strong*.*” (3)*
**How should we share it?**	• Produce a video to share research with other families with MJD.
	• Share the Toolbox with:
	○ Families with MJD all over the world.
	○ Health professionals/support workers of families with MJD.
	○ Community stakeholders.
	*“I’m telling you this so it will spread around the world*, *so other people can copy us*.*” (1)*

### Phase 3 –build prototype

The research team built the Toolbox prototype (spiral bound A3 book) based on findings of Phase 2. Panel members provided feedback on design and content and expressed that users of the Toolbox would need additional information to understand how this research and workbook developed. They also felt the Toolbox should be available for everyone to read (i.e., families with MJD and the general community) to improve understanding of MJD. The results of Phase 3 and Toolbox prototype outline are in Table 6.

*“…People need to know about this*…*so our communities understand this MJD” (12)*

**Table 6:** Staying Strong Toolbox prototype.

Book Section	Content Description
About This Book	• How this research happened
	• Why families wanted it to happen
Where Did This Book Come From?	• People involved in this research
	• How the research happened
	• Why this research is important
Why is it Good to Keep Yourself Strong Inside and Outside?	• Reasons to keep walking and moving around
	○ From families with MJD in the Top End
	○ From international MJD researchers
Staying Strong Inside and Outside	• Overview of the ‘Staying Strong Toolbox’
Exercising Your Body	• Tools for each functional level
Going Country	• Tools for each functional level
Searching for Good Medicine	• Tools for each functional level
Keeping Yourself Happy	• Tools for each functional level
Families Helping Each Other	• Tools for each functional level
Something Important to do	• Tools for each functional level
What will I do to Stay Stronger for Longer: Worksheet	• What really matters to me?
	• What do I want to work on in the next 4 weeks?
	○ How good am I at this today?
	○ What do I need to do to stay strong at this?
	○ What am I doing already to help with this?
	○ What help will I need?
	○ Who will I get help from?
Tools I Will Use to Keep Myself Strong	• All the tools I will use from each domain
My Plan Worksheet	• A planning sheet for individuals/their supporters
	• Demonstrate how to fit tools in everyday life

On completion of Phase 3, all panel members expressed that a trial would be required to determine if any further information should be added or removed

*“I want to see this happen now…we should do it so we can show people now that this works*…*” (6)*

In preparation for a subsequent trial, the research team created a section at the end of the Toolbox book, titled ‘Tools I Will Use to Keep Myself Strong’. This provided a section for all tools selected to be collated on one page to assist with planning on how to fit those activities into everyday life.

### Phase 4 –validation of prototype

Four panel members with MJD trialled the Toolbox workbook prototype to validate it for use in the planned pilot trial (Phase 6). Panel members worked through each section of the Toolbox, relevant to their level of function. Each panel member ticked, with a pen, a range of tools (activities) they would wish to do as part of their Toolbox program. Selections were collated at the end of the book in the ‘Tools I Will Use to Keep Myself Strong’ section. Two panel members went through this process while JC and JL observed. One panel member went through the same process with their partner present only, and one did so independently without support. Once completed, all panel members came together with JC, JL, GwL or BD, discussed what they had selected and explained the goals they wished to work on, verifying that the Toolbox prototype could be used as it was intended.

All who trialled Toolbox prototype felt it was useful to help think of ways to stay strong and felt positive about the process of selecting tools that suited them best. They identified irrelevant or repetitive tools, where text needed to be enlarged or where wording could be improved. They were able to easily identify goals to work on and score themselves as to how well they could complete the task at that time.

*“Ticking the boxes was good. Doing it our way*. *You see this Toolbox [of mine], it is good for making yourself strong and we need to do this…” (6)*

*“I like the way we did that Toolbox*, *because you have to see what you are doing first and tick it in that Toolbox, and then you go and do those things…” (3)*

*“Yeah like we made all the decisions about what we were going to do*… *it felt good like putting those things on the paper… and we gotta do those things…” (4)*

All four panel members expressed that individuals requiring support to select tools in the Toolbox workbook would need those supporters to understand their background and what mattered to them. Trusted relationships with those supporters was considered to be vital. As a result, a section titled ‘My Story’ was added at the beginning of the Toolbox prototype. This section provided a space for individuals using the workbook to explain 1) their story, including their personal and environmental factors, 2) whether they were strong, wobbly or using a wheelchair, 3) what was important to them, 4) what they enjoy doing and 5) what was challenging for them to do. With these additions, participants felt the Toolbox would be ready for trial as a program. Home programs were compiled collaboratively with each panel member with MJD, using the ‘My Plan Worksheet’ section, based on tools they selected in this validation phase whilst awaiting trial commencement. The full Staying Strong Toolbox prototype developed prior to trial commencement is included as a S1 File. Further modifications are anticipated after the trial is complete.

### Phase 5 –translation and celebration

At the completion of Phase 4, panel members felt the time was right for a community get-together with panel members, their families and anyone else interested, to inform the community of the work that had occurred and to celebrate what had been achieved.

*“I feel proud to see my families doing this work*. *It’s important. I want them to stay strong” (9)*

*“It was a hard job for us*, *and hard for me, telling these stories, thinking of my families, but I am proud…” (4)*

Panel members felt that the best way to share the Toolbox with other families and communities with MJD around the world was to produce a video to outline the research process, how the Toolbox could be used, and how well it worked for them. Funding was secured and a video was produced.

*“We really wanted to make this… so all of you can see and know about these things*. *When lots of people see it, in lots of different places, they can know about this weakness that people are carrying. We put all these stories together. So then we will all know about it. Us Aboriginal and non-Aboriginal people, and others in lots of different places and gathered them into one. This is the one for them (the Toolbox) to make themselves strong…” (13)*

## Discussion

The Staying Strong Toolbox forms a physical activity and lifestyle program, grounded in the experiences of families with MJD and current MJD research from around the world. The Toolbox enables individuals with MJD to select from a range of activities that will suit them, designed to maximise their physical and psychosocial well-being. Amidst recommendations for person-centred, meaningful, satisfying and challenging physical activity programs [15], this appears to be the first of its kind for people with ataxia [33]. Programs tailored to the individual for a range of other neurodegenerative conditions are also typically prescribed by a health professional, rather than user led or goal driven [33, 54–58].

The co-design process used to develop the Staying Strong Toolbox could be used as a guide for program development among individuals living with other ataxias and neurodegenerative diseases. The process allowed individuals living with MJD to contribute to improvements in care that they wanted to see happen [59, 60]. The co-design process allowed cultural responsiveness, kept decision-making power with the ultimate end users of the program and fostered their personal commitment to implementation [31, 61, 62]. Co-design has been used widely in software development and health service model development [30, 31, 63, 64] but less commonly in the development of physical activity interventions or rehabilitation programs [65, 66]. However, the approach has become recognised as more likely to produce feasible successfully implemented programs [30, 67, 68].

In line with successful co-design studies [61], effective collaboration to develop a person-centred program is dependent on the investment of time, flexibility and responsiveness to what works for the end user. In this study, families were able to lead the process, which was designed to fit within their lives, rather than add burden. Panel members were able to participate in their first language (e.g., Anindilyakwa or Kriol) which further fostered strong community participation in this research [69], but lacks emphasis in the co-design literature [70, 71]. Trusted community researchers led the work in each community, which ensured panel members could express themselves fully and consolidate their commitment to the process. The co-design process strengthened existing partnerships between local community researchers, the non-Aboriginal researcher and families with MJD [31].

Flexibility throughout the process ensured all panel members could contribute meaningfully [72]. Meetings were conducted at times that best suited those involved, commonly in the morning or late afternoon or evening, when temperatures were cooler, people were most active, and families were together. Much of the work would not have been possible if squeezed within a ‘9 to 5’ framework, which to our knowledge has not been discussed in the literature. The impact of providing ways for panel members to participate flexibly is evidenced by 100 percent panel member participation in every co-design phase. Research funding as well as logistical support from the MJD Foundation allowed this to be possible and was critical to the success of the project, particularly in relation to flexibility around funding and reporting timeframes as the project evolved [70]. Other studies have highlighted the difficulties in securing funding and ethics approvals for co-design projects, where truly responsive projects evolve over time, and specific details on what will occur and how long it will take prior to project commencement is a guess at best [61].

The Toolbox, created through a co-design process, is a source of pride to the community that has developed it, and who advocate for it to be implemented long term and shared widely. Consistent with an EBCD approach, the next step is to pilot use of the Toolbox to demonstrate feasibility and acceptability in helping families with MJD to keep walking and moving around.

### Limitations

The Staying Strong Toolbox may not be transferrable to other communities in its current form but provides a matrix adaptable for use in most parts of the world through a similar co-design process. The number of MJD Expert Panel members may be considered small compared to similar co-design studies, but was reflective of the remote setting [61]. Project costs may be considered high, although co-design studies rarely report this detail, so it is difficult to draw comparisons [73]. Genuine collaborative co-design in remote Aboriginal communities takes time [31] and can be costly, with long distance remote area travel, accommodation costs and wages. True flexibility and responsiveness to work alongside families in each community may have posed a limitation for some researchers who may not be local to a community, or who lack access to accommodation, transport or flexibility to work throughout the day and evening for extended periods of time [74]. However, the ability and willingness to be flexible worked to be a strength in this study.

While not a proficient speaker of Anindilyakwa or Kriol, the non-Aboriginal researcher’s efforts to learn and converse in the languages spoken in each community were taken seriously and observed to have had a strong impact on partnerships, relationships and trust [75]. Zoom and telephone provided an easy, flexible, cost effective way to maintain communication between visits.

### Implications for practice

The Toolbox provides a resource that requires no training and can be used by families with MJD, other ataxias, as well as by health professionals who are supporting them to help individuals keep walking and moving around. Individuals with MJD can use the Toolbox either on their own or with the support of their families and health professionals. Health professionals can read through the Toolbox with the individual to understand the tools they have selected and what is important to them. This process can help understand and facilitate the ways in which the individual may operationalise those tools within their everyday life. The Toolbox provides a guide for individuals to develop their own person-centred program to keep themselves walking and moving around in a way specifically important to them. While a pilot of the Toolbox is planned as the final stage of co-design, the process described may be useful for other communities and researchers who wish to co-design a similar person-centred program. Although co-design takes time, the investment is likely to add to the benefits in physical and psychosocial well-being of families with MJD and program sustainability into the future.

## Conclusion

The Staying Strong Toolbox was co-designed with and for Aboriginal families with MJD living on Groote Eylandt and in Ngukurr, based on their experiences and informed by knowledge gained from MJD research worldwide. The co-design process produced a meaningful physical activity and lifestyle program designed for families with MJD, by families with MJD. The Staying Strong Toolbox and co-design process could be used by other individuals with MJD, or individuals with other ataxias or neurodegenerative diseases more broadly. The next step is to conduct a pilot study to determine the feasibility and acceptability of the Toolbox.

## Supporting information

S1 File Staying Strong Toolbox prototype. (DOCX)
