# Supplementary material for: Staying Strong Toolbox: Co-design of a physical activity and lifestyle program for Aboriginal families with Machado-Joseph disease in the Top End of Australia
Source: PLoS One. 2021 Feb 5;16(2):e0244311. doi: 10.1371/journal.pone.0244311 (PMC7864457; doi:10.1371/journal.pone.0244311)
Supplement: S1 File — (DOCX) [file pone.0244311.s001.docx]

# S1 File – Staying Strong Toolbox Prototype

Staying Strong Toolbox Prototype

<https://doi.org/10.6084/m9.figshare.13370228.v1>
